# Cyclosporin A attenuating morphine tolerance through inhibiting NO/ERK signaling pathway in human glioblastoma cell line: the involvement of calcineurin

**DOI:** 10.17179/excli2018-1693

**Published:** 2018-11-12

**Authors:** Asma Rashki, Faiza Mumtaz, Farahnaz Jazayeri, Amir Shadboorestan, Jamileh Esmaeili, Shahram Ejtemaei Mehr, Mohammad Hossein Ghahremani, Ahmad Reza Dehpour

**Affiliations:** 1 Experimental Medicine Research Center, Tehran University of Medical Sciences, Tehran, Iran; 2 Department of Pharmacology, School of Medicine, Tehran University of Medical Sciences, Tehran, Iran; 3 Department of Toxicology and Pharmacology, Faculty of Pharmacy, Tehran University of Medical Sciences, Tehran, Iran

**Keywords:** Cyclosporin A, morphine, tolerance, nitric oxide, T98G cells, extracellular signal-regulated kinases

## Abstract

Cyclosporin A (CsA) is known to have an immunosuppressive action. However, it is also attracting attention due to its effects on the nervous system, such as inhibiting the development and expression of morphine-induced tolerance and dependence through unknown mechanisms. It has been shown that CsA modulates the nitric oxide (NO) synthesis and extracellular signal-regulated kinases (ERK) activation, which are potentially involved in signaling pathways in morphine-induced tolerance in cellular models. Therefore, the current study was designed to evaluate the modulatory role of CsA on the MOR tolerance, by targeting the downstream signaling pathway of NO and ERK using an *in vitro* model. For this purpose, T98G cells were pretreated with CsA, calcineurin autoinhibitory peptide (CAIP), and N^G^-nitro-l-arginine methyl ester (L-NAME) 30 min before 18 h exposure to MOR. Then, we analyzed the intracellular cyclic adenosine monophosphate (cAMP) levels and also the expression of phosphorylated ERK and nitric oxide synthase (nNOS) proteins. Our results showed that CsA (1 nM, 10 nM, and 100 nM) and CAIP (50 µM) have significantly reduced cAMP and nitrite levels as compared to MOR-treated (2.5 µM) T98G cells. This clearly revealed the attenuation of MOR tolerance by CsA. The expression of nNOS and p-ERK proteins were down-regulated when the T98G cells were pretreated with CsA (1 nM, 10 nM, and 100 nM), CAIP (50 µM), and L-NAME (0.1 mM) as compared to MOR. In conclusion, the CsA pretreatment had a modulatory role in MOR-induced tolerance, which was possibly mediated through NO/ERK signaling pathway.

## Introduction

Cyclosporin A (CsA) is an immunosuppressant drug which mediates its action through binding with immunophilin. Through inhibiting calcineurin enzyme, the CsA and immunophilin complex blocks the signaling pathways of calcineurin/NFAT (Matsuda and Koyasu, 2000[42]). Calcineurin (CaN), also known as a calcium-regulated protein, is a Ca^2+^/CaM-dependent serine/threonine phosphatase, and causes the release and mobilization of Ca^2+^ from intracellular stores (Kipanyula et al., 2016[32]). Although CsA is an immunosuppressant, its modulatory role in the functions of the central nervous system (CNS) has been evaluated through its activity on the modulation of neurotransmitters release (Steiner et al., 1996[64]), its influence on the release of calcium from intracellular stores and neurotrophic activities (Snyder et al., 1998[61]). Moreover, its protective action against the neurotoxicity induced by nitric oxide (Trajković et al., 1999[67]) and glutamate (Ruiz et al., 2000[57]) serves as an evidence of the mentioned function. The inhibitory action of CsA on morphine-induced place conditioning (Langroudi et al., 2005[34]), on tolerance and dependence (Homayoun et al., 2002[25]; Mehr et al., 2003[44]) and also its antinociceptive effect (Homayoun et al., 2002[24]) through inhibiting the nitric oxide pathway have been studied previously.

Morphine (MOR) has been used efficiently in the management and treatment of chronic pain. However, the development and expression of tolerance limits the therapeutic efficacy of the drug (Pajohanfar et al., 2017[50]). The cellular and molecular mechanisms involved in morphine tolerance are not very clear and are linked to various signaling mechanisms, including an elevated activity of the adenylyl cyclase (AC) which increases the cyclic adenosine monophosphate (cAMP) levels (Christie, 2008[11]; Nestler, 2004[49]). Morphine-induced tolerance and dependence can possibly be prevented by reducing the upregulation of cAMP (Jamil et al., 2013[26]; Javadi et al., 2013[27]).

It has been reported that morphine tolerance can be induced by nitric oxide (NO) which is synthesized by nitric oxide synthase (NOS) (Mayer et al., 1999[43]), so that increased NOS activity accelerates the induction of morphine tolerance. Furthermore, NOS inhibitors attenuate this effect (Dambisya and Lee, 1996[12]; Hassanipour et al., 2018[21]; Khan et al., 2017[31]; Rahmati et al., 2017[53]). NOS is activated by the influx of Ca^++^ ions that can be linked to nNOS which is an isoform of NOS and modulates morphine tolerance (Heinzen and Pollack, 2004[23]). It has been reported that nNOS inhibitors have reversed morphine tolerance (Liu et al., 2006[37]). Extracellular signal-regulated kinases (ERK) is one of the gene products of mitogen-activated protein kinases (MAPK) pathway (Raman et al., 2007[54]). Inhibiting morphine-induced ERK activity and ERK phosphorylation could be used as a signaling mechanism to attenuate morphine tolerance (Hawes et al., 2008[22]; Macey et al., 2009[39]). Moreover, chronic morphine mediates the increased level of nNOS which can be modulated by the inhibition of the ERK pathway (Wang et al., 2011[69]).

As far as CsA is concerned, AC activation is attenuated by CsA (Banafshe et al., 2007[4]). The catalytic activity of nNOS is reduced by cyclosporine A in the nervous system through the modulation of calcineurin-dependent dephosphorylation of nNOS which subsequently inhibits NO release (Rao et al., 1996[55]; Snyder et al., 1998[61]). It has been reported that CsA decreases the activity and expression of nNOS (Diaz-Ruiz et al., 2005[14]). On the other hand, CsA can inhibit ERK activation (Gary-Gouy et al., 2006[19]) and cytokine-induced phosphorylation of ERK (Doller et al., 2007[16]) as described in some previous researches.

According to what was discussed above, CsA seems to have modulatory effects on cAMP, nitric oxide, and ERK pathways. Therefore, in the current study, we aim to uncover the underlying potential mechanisms of CsA in reducing morphine-induced tolerance by investigating the NO/ERK pathway through conducting different molecular studies *in vitro*. We also attempt to study the possible involvement of calcineurin under these effects.

## Materials and Methods

### Drugs and reagents

In the present study, the used drugs and reagents were: Cyclosporin A, Morphine hydrochloride, N (G)-nitro-L-arginine methyl ester (L-NAME), vanadium, sulfanilamide, sodium nitrite (NaNO2), 3-(4,5-Dimethylthiazol-2-yl)-2,5-diphenyltetrazolium bromide (MTT), N-(1-naphthyl) ethylenediamine and trypsin-EDTA. All of these drugs and reagents were acquired from Sigma (St. Louis, MO, USA). High glucose Dulbecco's modified Eagle's medium (DMEM), fetal bovine serum (FBS), and penicillin/streptomycin were obtained from Biosera (Vienna, Austria). The other used materials included calcineurin auto-inhibitory peptide (CAIP; Tocris Bioscience, UK), cAMP ELISA Kit (Aviscera Bioscience, USA), and polyvinylidenedifluoride (PVDF) membrane (Millipore, Germany).

### Cell line and antibodies

In the present study, the used cell line was T98G human glioblastoma cell line (derived from a glioblastoma multiforme, WHO grade IV; Pasteur Institute, Tehran, Iran). The primary antibodies were nNOS (Neuronal nitric oxide synthase) monoclonal antibody, ß-actin antibody (Santa Cruz Biotechnology, USA), ERK (Extracellular signal-regulated kinases) antibody, and p-ERK (Phosphorylated ERK) antibody (Cell Signaling Technology, USA). The secondary antibody was horse-radish peroxidase (HRP)-conjugated antibody (BioRad, USA).

### Preparation of drugs and reagents

Morphine hydrochloride and L-NAME were prepared freshly in cell culture media. Dimethyl sulfoxide (DMSO; Merck, Germany) was used to prepare a stock solution of CsA. The DMSO (0.1 % v/v) was used in the experiments. To the morphine-treated cells, the same volume of DMSO was added as a vehicle. All the samples were prepared and diluted freshly before use. 

### Preparation and maintenance of cell culture

The cell culture of T98G human glioblastoma cell line is widely used for *in vitro* studies as it expresses high levels of the opioid receptors (Khan et al., 2017[31]; Lazarczyk et al., 2010[36]). The cells were grown in a DMEM medium supplemented with FBS (10 %) and penicillin/streptomycin (1 %). Then, they were kept in an incubator (37 ºC) and humidified (5 % CO_2_). The culture medium was changed every 24 h. Then, the cells were harvested by trypsin-EDTA (Ethylenediaminetetraacetic acid) solution (0.25 %). After seeding for 24 h, the cells were treated with morphine (2.5 µM) for 18 h (Avidor-Reiss et al., 1997[2]). Then, the cells were pretreated for 30 min with media containing cyclosporine (1 nM, 10 nM, and 100 nM), CAIP (10 µM and 50 µM), and L-NAME (0.01 mM, 0.1 mM, and 1mM) prior to chronic morphine treatment to evaluate their effects on morphine tolerance.

### Experimental protocols

#### Cell viability test (MTT assay)

In order to investigate the effect of drugs on the viability of cells, microculture tetrazolium test (MTT) assay was conducted as described previously (Khan et al., 2017[31], 2018[30]). To perform the assay, T98G cells were placed in 96-well culture plates for 24 h at a density of 2×10^4 ^cells per well. Then, the cells were exposed to media containing morphine (2.5 µM), morphine with CsA (1 nM, 10 nM, and 100 nM), morphine with CAIP (10 µM, 50 µM), and morphine with L-NAME (0.01 mM, 0.1 mM, and 1 mM) for 18 h. Next, the medium was removed and MTT solution (0.5 mg/ml/well) was added to each well and incubated in darkness for 3 h at 37 °C. Then, the solution was removed; the resulting blue formazan was solubilized in DMSO (100 µl), and its optical density was measured at 570 nm by using a microplate reader (Bio-Tek Synergy, US). By using serum-free growth medium as the blank, the viability of the cells was calculated by the following formula: 

(mean OD treatment [−blank])/(mean OD control [−blank]) × 100

#### Measurement of cAMP level

The cAMP level was analyzed in the current study based on the method described in the previous studies (Banafshe et al., 2007[4]; Javadi et al., 2013[27]). The cells were seeded in 96-well plates at a density of 2×10^5 ^cells per well, and incubated at 37 °C for 24 h. After removing the culture medium, the cells were exposed to drug treatments as listed for 18 h, and then, were treated with 1 μM forskolin (FSK) for 10 min (Rhee et al., 2000[56]). Then after washing, the cells were lysed with 0.1 M HCl (500 mL/well) for 15 min and centrifuged at 600 × g at 4 °C and their supernatant was collected to measure cAMP levels using direct cyclic AMP ELISA (Enzyme-linked immunosorbent assay) Kit (Aviscera Bioscience, USA).

#### Nitrite assay

In order to measure the amount of nitrite oxide in the drug-treated cells, nitrite assay by Griess reaction was performed as described previously (Hassanipour et al., 2018[21]; Khan et al., 2017[31]). To measure the nitrite levels, 100 μL of the supernatant of each sample was transferred into 96-well microplates and sulfanilamide solution (50 μL), and vanadium (10 μL) were added to each sample and incubated at room temperature for 5 min. After the incubation, each sample was mixed with N-(1-naphthyl) ethylenediamine (50 μL) solution (0.1 % in water), and again incubated for 30 min under the reduced light conditions. Afterward, by using a microplate reader (Bio-Tek Synergy HT, US), all the samples were analyzed at 540 nm at room temperature. A nitrite standard of 0.1 M NaNO_2_ in water was used to analyze nitrite levels for each sample.

#### Western blot analysis

In order to perform this analysis, 6-well microplates were used to seed the T98G cells at a density of 3×10^5 ^cells per well for 24 h. After removing the culture medium, the T98G cells were treated with morphine and all the treatments mentioned above for 18 h at effective concentrations on the basis of the cAMP assay. After applying the treatments, the next step was to prepare total protein extracts by using lysis buffer (Tris buffer 62.5 mM at pH 6.8, dithiothreitol 50 Mm, bromophenol blue 0.25 % (W/V), and glycerol 1 %). The samples were resolved on a SDS-PAGE gel (10 %), which were then moved onto the PVDF (Polyvinylidenedifluoride) membrane. For a period of 2 h, the PVDF membranes were blocked by skim milk (5 %) and incubated by primary β-actin, ERK, p-ERK, and nNOS antibodies overnight. The blots were probed with β-actin as the loading control. The membranes were then washed with TBST (a mixture of Tris-buffered saline and polysorbate 20) followed by the incubation with horse-radish peroxide (HRP)-conjugated secondary antibodies for 1 h at room temperature. A BM chemiluminescence detection system (Roche, Germany) was used to develop the blots. The optical density of each band was quantified by using Image J (an open source image processing software).

### Statistical analysis

In our study, one-way analysis of variance (ANOVA) followed by Tukey's post-hoc test was applied where appropriate to analyze the statistical difference among various groups by using GraphPad Prism 7 (San Diego, CA, USA). Herein, the data were presented as mean ± SEM, and the probability (p) value of less than 0.05 (p< 0.05) was considered statistically significant in all the experiments. 

## Results

### The effects of the treatments on cell proliferation 

**Figure 1:**
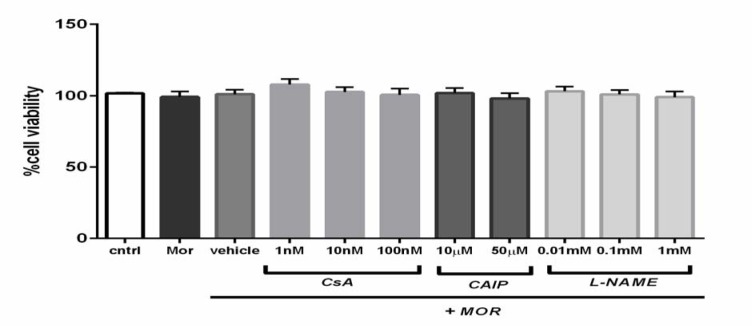
The survival of the T98G cells was evaluated by MTT assay. None of the studied drugs exhibited any toxicity on the viability of the T98G cells after 18 h compared to the control, vehicle (comprised of 0.1 % DMSO (V/V)), and MOR. The results were presented as mean ± SEM of three independent experiments conducted in triplicate. CsA (Cyclosporin A); MOR (Morphine); CAIP (Calcineurin auto-inhibitory peptide); L-NAME (NG-nitro-l-arginine methyl ester)

In order to nullify the cytotoxic effect of the used drugs, MTT assay was conducted in advance. The cells were incubated with culture medium, MOR (2.5 μM), and the combination of MOR with CsA (1 nM, 10 nM, and 100 nM), with CAIP (10 µM and 50 µM) and with L-NAME (0.01 mM, 0.1 mM, and 1 mM) for 18 h. The results of the assay indicated that the studied compounds had no toxic effects on the T98G cells compared to the untreated, morphine-treated, and the vehicle-treated cells (Figure 1(Fig. 1)).

### The effects of treatments on cAMP (cyclic adenosine monophosphate) levels

#### The effect of chronic morphine on cAMP levels

**Figure 2:**
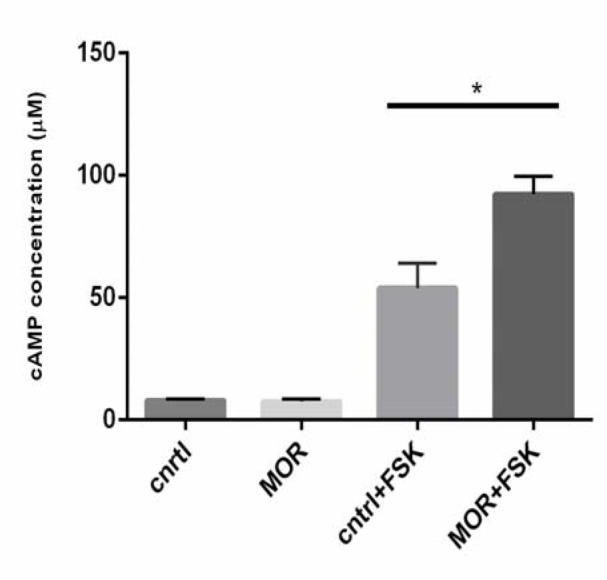
Prolonged effect of MOR on cAMP levels with and without forskolin. The MOR (2.5 µM for 18 h) treatment up-regulated cAMP (about 1.7 folds) in the T98G cells. All the data were expressed as mean ± SEM of three experiments conducted in duplicate. *p˂0.05 as compared to control+FSK

Herein, cAMP levels in the T98G cells were measured in the presence of MOR (2.5 µM for 18 h). The cAMP levels in the T98G cells were indiscernible due to their instability in our pilot study, i.e. in both control and morphine-treated groups. Therefore, we stimulated the cells with the application of forskolin (FSK; 1 µM) for 10 min before harvesting our cells, because FSK is used to study the effects of the inhibition or activation of cAMP (Alasbahi and Melzig, 2012[1]). The results showed that FSK has significantly enhanced the level of cAMP in both control and chronic morphine-treated groups. The results also indicated that morphine increases the cAMP levels, compared to the control group after adding forskolin to both groups (*p˂0.05, Figure 2(Fig. 2)).

#### The effects of CsA and CAIP on cAMP elevation

**Figure 3:**
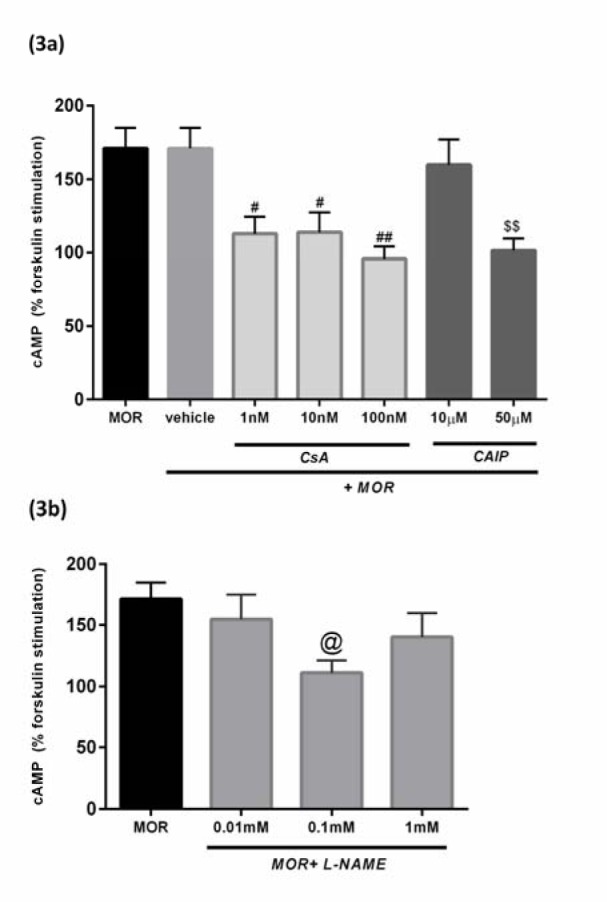
Measurement of cAMP levels (µM). a: Pretreatment with CsA (1 nM, 10 nM, and 100 nM) and CAIP (50 µM) inhibited the FSK-stimulated cAMP in MOR-treated cells. b: Pretreatment of cells with L-NAME (0.1 Mm) significantly reduced the FSK-mediated cAMP concentration as compared to the MOR-treated cells. Concentrations of cAMP were determined by ELISA kit, and the data were presented as a percentage of FSK-stimulated cAMP. All the data were presented as mean ± SEM of three experiments conducted in duplicate. Multiple comparisons among groups were performed by one-way analysis of variance (ANOVA), followed by Tukey's post-hoc test. #p< 0.05 and ## p<0.01 versus the vehicle-treated group and $$p<0.01, @p<0.05 versus the morphine-treated group. The vehicle was comprised of 0.1 % DMSO (v/v) and MOR.

To study the effect of the CsA on the cAMP accumulation induced by MOR, pretreatment of cells for 30 min with CsA (1 nM, 10 nM, and 100 nM) was done, before treating them with MOR (2.5 µM for 18 h). The results showed that CsA significantly inhibited the FSK-stimulated cAMP concentration compared to the vehicle-treated group (#p< 0.05, ## p<0.01, Figure 3a(Fig. 3)). The inhibitory effect of CsA on the morphine-induced cAMP overshoot is dependent on its calcineurin inhibitory property which was confirmed by using CAIP. To determine the mentioned effect, the pre-incubation of the cells with CAIP (50 µM) for 30 min before MOR treatment significantly blocked the MOR-induced increase of cAMP in the T98G cells, compared to the morphine-treated group ($$p<0.01, Figure 3a(Fig. 3)). 

#### The effect of L-NAME on cAMP elevation

In order to examine the possible correlations between the cAMP levels induced by the chronic morphine exposure and the activity of NOS enzyme in the cells, L-NAME (0.1 mM) was used which significantly inhibited the MOR-induced (2.5 µM for 18 h) increase in the cAMP levels (@p<0.05, Figure 3b(Fig. 3)).

### The effects of treatments on nitrite levels

#### The effect of chronic morphine on nitrite levels

**Figure 4:**
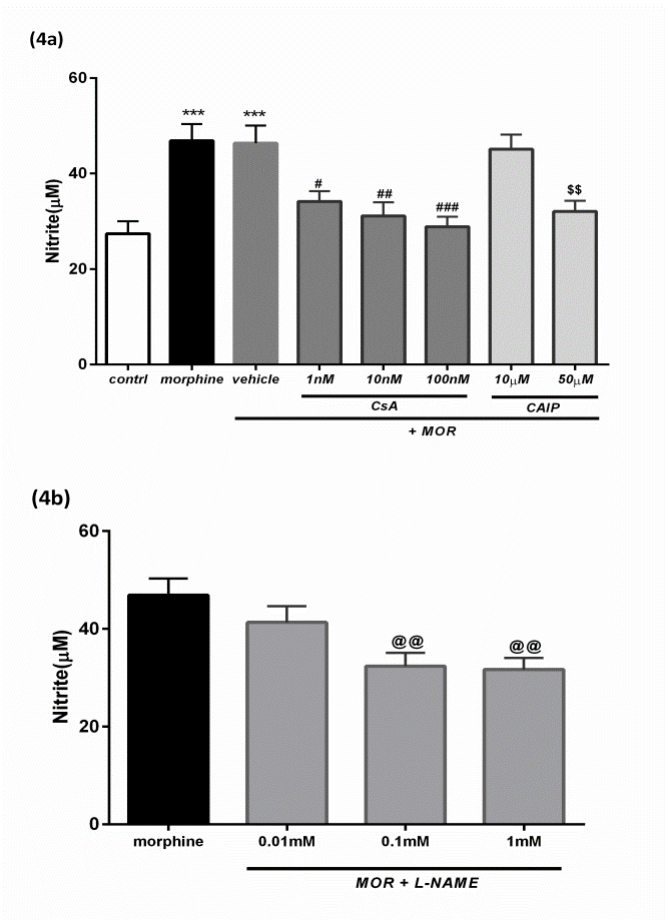
Measurement of nitrite levels (µM). a: Nitrite levels (µM) significantly increased after morphine treatment (2.5 µM for 18 h) as compared to the control. Pretreatment with CsA (1 nM, 10 nM, and 100 nM) and CAIP (50 µM) significantly inhibited nitrite production induced by MOR. b: Pretreatment with L-NAME (0.1 mM and 1 mM) significantly inhibited the nitrite production induced by MOR. All data were presented as mean ± SEM of three experiments conducted in triplicate. Multiple comparisons among the groups were performed by one-way analysis of variance (ANOVA) followed by Tukey's post-hoc test. ***p<0.001 versus control group, #p< 0.05, ## p<0.01 and ###p<0.001 versus vehicle-treated group, $$p<0.01, @@p<0.01 versus morphine-treated group. The vehicle was comprised of 0.1 % DMSO (v/v) and MOR.

In order to analyze the effect of chronic morphine (2.5 μM for 18 h) exposure on the NOS (nitric oxide synthase) activity and NO (nitric oxide) level, the T98G cells were treated with and without morphine. The results showed that morphine significantly increased the nitrite formation in the cells as compared to the control (***p<0.001, 1.7 folds, Figure 4a(Fig. 4)). 

#### The effects of CsA and CAIP on nitrite levels

Pre-treatment of the cells for 30 min with CsA (1 nM, 10 nM, and 100 nM) before treating them with chronic MOR, significantly diminished the effects of morphine on the nitrite production as compared to the vehicle-treated group (#p<0.05, ##p<0.01 and ###p<0.001, Figure 4a(Fig. 4)). Similar results were observed when the cells were pretreated for 30 min with CAIP (50 µM) before adding chronic MOR to the T98G cells ($$ p<0.01, Figure 4a(Fig. 4)).

#### The effect of L-NAME on nitrite levels

In this study, the morphine-induced stimulation of the NO formation was significantly inhibited by the pretreatment of the cells with L-NAME (0.1 mM and 1 mM) for 30 min before chronic morphine incubation (2.5 µM for 18 h) in the T98G cells (@@p<0.01, Figure 4b(Fig. 4))

### Cyclosporin A modifying ERK1/2(Extracellular Signal-Regulated Kinases) pathway in the T98G cells

#### The effect of chronic morphine on the phosphorylation of ERK1/2

**Figure 5:**
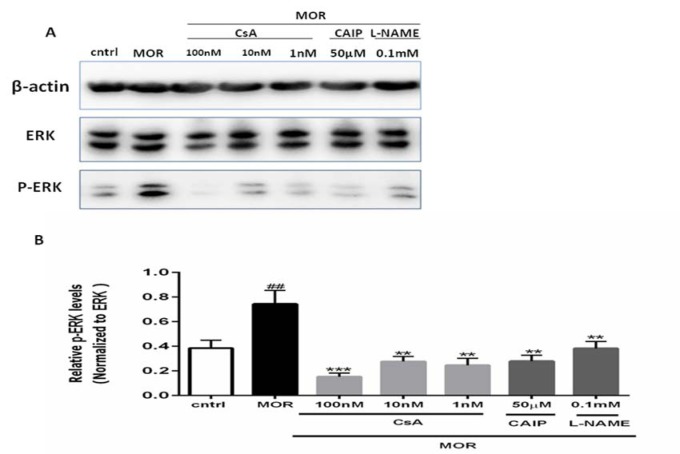
The effect of CsA on phosphorylation of ERK: The representative immunoblots for p-ERK1/2, ERK1/2, and β-actin are presented, in which the band intensity of p-ERK was normalized as compared to ERK1/2. The densitometric analysis of the immunoblot was conducted by using Image J software and the results were presented as a percentage of control. The results showed that chronic MOR (2.5 µM for 18 h) induced phosphorylation of ERK1/2 in the T98G cells, while the pretreatment of cells with CsA (1 nM, 10 nM, and 100 nM), CAIP (50 µM), and L-NAME (0.1mM) suppressed the stimulatory effect of chronic MOR treatment on phosphorylation of ERK1/2. All the data were presented as mean ± SEM of three experiments, conducted in duplicate. Multiple comparisons among groups were performed by one-way analysis of variance (ANOVA), followed by Tukey's post-hoc test. ##p<0.01 in comparison to the control, ***p<0.001, **p<0.01 in comparison to MOR.

The extracellular signal-regulated kinase (ERK) signaling is involved in developing morphine tolerance (Berta et al., 2013[6]). In our study, we observed that the chronic exposure of morphine increased the phosphorylation of ERK after incubation of the T98G cells with morphine (2.5 µM) for 18 h as shown in Figure 5(Fig. 5) (##p<0.01).

#### The effects of CsA, CAIP, and L-NAME on the phosphorylation of ERK1/2

The Western blot analysis for β-actin, ERK, and p-ERK proteins was performed by using their specific antibodies as shown in Figure 5(Fig. 5). For this purpose, the cells were pre-incubated for 30 min with CsA (1 nM, 10 nM, and 100 nM) before MOR treatment. The chronic morphine-mediated phosphorylation of ERK1/2 was significantly inhibited by CsA (***p<0.001, **p<0.01). The similar results were observed with the pretreatment of cells for 30 min with CAIP (50 µM) and L-NAME (0.1 mM) before MOR treatment (**p<0.01). 

### Cyclosporin A modifying nNOS (Neuronal Nitric Oxide Synthase) protein expression in the T98G cells

#### The effect of chronic morphine on the expression of nNOS protein

**Figure 6:**
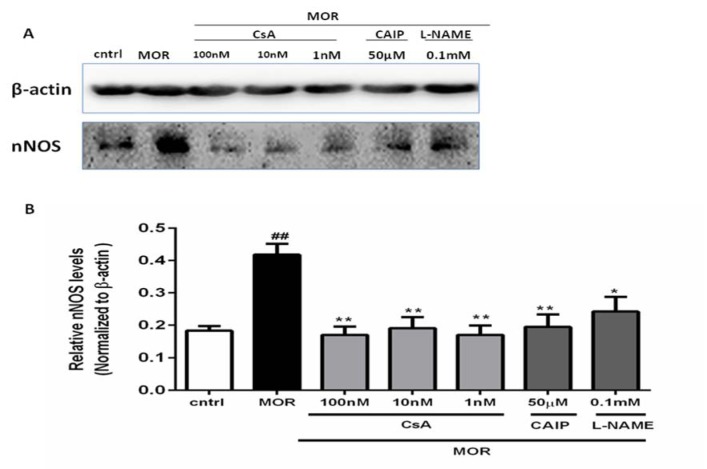
The effect of CsA on the protein expression of nNOS: The representative immunoblots for nNOS and β-actin are shown in the figure, and densitometric analysis of the immunoblot was conducted by using Image J software and the results were presented as a percentage of the control. The expression of nNOS was enhanced in the T98G cells exposed to chronic MOR (2.5 µM for 18 h), while pretreatment of cells with CsA (1 nM, 10 nM, and 100 nM), CAIP (50 µM), and L-NAME (0.1 mM) suppressed the stimulatory effect of chronic MOR treatment on nNOS protein expression. All data were presented as mean ± SEM of three experiments conducted in duplicate. Multiple comparisons among groups were performed by one-way analysis of variance (ANOVA), followed by Tukey's post-hoc test. ##p<0.01 in comparison to the control, **p<0.01, *p<0.05 in comparison to MOR.

The expression of nNOS protein was up-regulated after exposing the cells to morphine (2.5 µM for 18 h) (##p<0.01, Figure 6(Fig. 6)), which suggests that nNOS protein expression might be involved in the development of the morphine tolerance in the T98G cells.

#### The effects of CsA, CAIP, and L-NAME on the expression of nNOS protein

The results of the current study have already shown that the pretreatment of cells with CsA inhibited the formation of chronic morphine-induced NO (Figure 4a(Fig. 4)). Western blot analysis was conducted to indicate whether the effect of CsA was at least partially mediated through modifying the expression of nNOS. The cells were pretreated for 30 min with CsA (1 nM, 10 nM, and 100 nM) prior to morphine treatment (2.5 µM for 18 h). As shown in Figure 6(Fig. 6), the CsA significantly inhibited the morphine-mediated nNOS expression in the T98G cells (**p<0.01). Similar results were observed with the pretreatment of cells with CAIP (50 µM) and L-NAME (0.1 mM), which indicated that the specific inhibition of calcineurin and the inhibition of NOS can alter morphine tolerance in the T98G cells by down-regulating nNOS protein expression (*p<0.05,**p<0.01).

## Discussion

The current study demonstrated that CsA attenuates MOR-induced tolerance through extracellular signal-regulated kinases (ERK) pathway, which is likely a result of the significant decrease of nitric oxide (NO) synthesis in morphine-dependent T98G cells.

A state of adaptation in which the effect of the drug is diminished with the passage of time due to prolonged exposure is defined as tolerance (Taylor and Fleming, 2001[65]). It is very obvious that morphine is the best therapeutic choice for severe pain (Ballantyne and Mao, 2003[3]); numerous clinical and preclinical investigations have revealed that tolerance to the anti-nociceptive effects of morphine is the main concern which limits its clinical use (Bekhit, 2010[5]; Pajohanfar et al., 2017[50]). Studies have reported that calcineurin also plays a role in the neuronal adaptations mediated by MOR (Biala et al., 2005[7]). Furthermore, the activation of calcineurin by morphine has already been reported (Kam et al., 2010[28]).

Cyclosporin A which was initially isolated from fungi, is a powerful immunosuppressant (Smith, 2017[60]). To exert this effect, it inhibits two forms of cytoplasmic targets, including calcium/calmodulin-dependent protein serine/threonine phosphatase which is called calcineurin, and peptidyl-prolylcis-trans-isomerase (PPIase) (Terada et al. 2003[66]). Thus, by binding itself to immunphilins, which are protein receptors of CsA, Cyclosporin A inhibits the phosphatase activity of CaN (Matsuda and Koyasu, 2000[42]). Interestingly, it has been shown that these immunophilins are fifty folds more prevalent in the central nervous system (CNS) as compared to the immunity system (Steiner et al., 1992[63]). They are called neuroimmunophilins and mediate neuronal activity (Gold, 2000[20]). Dougherty et al. reported for the first time in 1988 that opioid-induced dependence was attenuated by CsA (Dougherty et al., 1988[18]). Furthermore, CsA attenuated the morphine-induced place conditioning (Langroudi et al., 2005[34]), and also, the development and expression of the tolerance and dependence (Homayoun et al., 2002[24]) by inhibiting NO production. Moreover, it has been reported that the neural effects of immunophilin ligands are considerable for the inhibition of morphine-induced tolerance besides their immunologic actions (Mehr et al., 2003[44]). 

The adenylyl cyclase (AC), enzyme catalyzes the formation of cAMP and modifications in cAMP signaling, play a crucial role in the development of morphine tolerance (Christie, 2008[11]; Nestler, 2004[49]). An increased level of cAMP can be considered as a major mechanism in causing morphine tolerance as described by many researchers in their *in vitro* studies, including EcR293 cell line (Zhao et al., 2006[74]), HEK 293 cell line (Tso and Wong, 2001[68]), CHO cell line (Yue et al., 2006[73]), and human neuroblastoma SK-N-SH cell line (Jamil et al. 2013[26]). The AC isoenzyme shows its sensitization for different kinds of stimulators, like forskolin (FSK) through its binding with Raf-1 (Ding et al., 2004[15]). Due to the same reason, in the current study, we added FSK to the cells and observed that chronic MOR induced an increase in cAMP levels in the T98G cells (Figure 2(Fig. 2)). It has been previously reported that CsA can attenuate the development of cannabinoid-induced up-regulation of cAMP in human astrocytoma cells (Banafshe et al., 2007[4]), and in cultured neurons of the hippocampus (Chan et al., 2005[9]). In this study, the possible involvement of CsA on the modulation of morphine tolerance was determined through its effect on the cAMP levels, due to the fact that CsA significantly attenuated the chronic MOR-induced cAMP levels in the T98G cells (Figure 3(Fig. 3)). 

Nitric oxide (NO) is one of the main mediators involved in the adaptations to chronic morphine-induced tolerance as the inhibition of nitric oxide synthase (NOS) activity results in the attenuation of morphine-induced tolerance (Hassanipour et al., 2018[21]; Heinzen and Pollack, 2004[23]; Naidu et al., 2003[48]; Raghavendra et al., 2000[52]). In one study, thalidomide treatment reversed morphine tolerance by decreasing NOS and nitric oxide pathway in T98G cells (Khan et al., 2017[31]). In the present study, NO levels were significantly increased in the morphine-treated group. CsA inhibits NO synthesis in glioma C6 cell line (Trajković et al., 1999[67]). Moreover, this effect of the CsA on the nitrite production, is mediated through the inhibition of CaN, because CaN dephosphorylates NOS and up-regulates its activity (Kaminska et al., 2004[29]). In contrast, CsA increases NOS phosphorylation and inhibits its enzyme activity (Dawson et al., 1993[13]). The results of the present study showed that CsA significantly reversed the effect of morphine-induced tolerance by decreasing the levels of NO in the T98G cells (Figure 4(Fig. 4)). 

Previous studies have reported that a sub-family of mitogen-activated protein kinases (MAPK) is extracellular signal-regulated protein kinases (ERK) which is involved in morphine tolerance (Hawes et al., 2008[22]; Macey et al., 2009[39]; Polakiewicz et al., 1998[51]). In some studies, MOR mediated the phosphorylation of MAPK in dorsal root ganglion neurons (Ma et al., 2001[38]) and ERK (Chen et al., 2008[10]). Furthermore, some other studies concluded that the expression of p-ERK was increased by MOR in cultured astrocytes (Berta et al., 2013[6]), and in the primary culture of striatal neurons (Macey et al., 2006[40]). The MOR-induced tolerance could be attenuated by ERK inhibitors (Wang et al., 2009[70]). Moreover, morphine tolerance could be reduced by miR-365 by inhibiting ERK signaling pathway (Wu et al., 2018[72]). In the current study, the exposure of T98G cells to chronic morphine significantly up-regulated the phosphorylation of ERK1/2 as compared to the control. It has been reported that in rat glomerular mesangial cells (MC), CsA attenuated the cytokine-mediated phosphorylation of ERK and JNK (Doller et al., 2007[16]) and reversed the phosphorylation of ERK1/2 in anti-IgM-activated Daudi B-cells by diverting Raf from the MAPK pathway (Gary-Gouy et al., 2006[19]). However, previous studies reported that CaN led to calcium-mediated activation and phosphorylation of MAPK and ERK (Dougherty et al., 2009[17]; Molkentin, 2004[46]), and this CaN-dependent activation of ERK1/2 was blocked by CsA in cultured cardiomyocytes (Zou et al., 2001[75]). Our study demonstrated that morphine-induced phosphorylation of ERK1/2 was significantly reversed by treating the cells with CsA (Figure 5(Fig. 5)). 

In response to physiological stimulation, NO is released by neuronal NOS (nNOS) which is a calcium/calmodulin-dependent enzyme (Larson et al., 2000[35]). The effect of morphine tolerance on NO production could be linked to nNOS. Therefore, nNOS has a dynamic role in regulating the expression of morphine-induced tolerance (Heinzen and Pollack, 2004[23]; Machelska et al., 1997[41]; Wong et al., 2000[71]). It was shown that in spinal microglial cells, the inhibition of nNOS modulated the morphine tolerance (Liu et al., 2006[37]). The results of our study revealed that the chronic treatment of cells with morphine significantly increased the expression of nNOS. CsA inhibits CaN and causes NOS phosphorylation (Snyder et al., 1998[61]). Therefore, nNOS is inhibited through phosphorylation, and CaN reverses this effect by dephosphorylation of NOS (Morioka et al., 1999[47]). Through CaN inhibition, CsA is able to diminish the catalytic activity of nNOS, which in turn inhibits the release of NO (Mehr et al., 2003[44]; Rao et al., 1996[55]; Sabatini et al., 1997[58]). On the other hand, in spinal cord injury, the nNOS-mediated NO production is attenuated by CsA as a result of the inhibition of the activity and the expression of nNOS (Diaz-Ruiz et al., 2005[14]). The treatment of cells with CsA significantly decreased the expression of nNOS as compared to morphine-treated cells (Figure 6(Fig. 6)).

**Figure 7:**
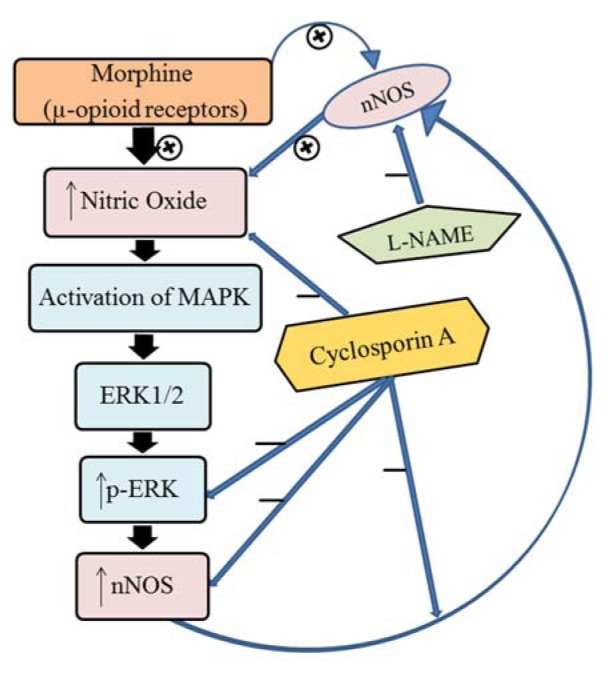
The role of CsA in the attenuation of morphine tolerance. ERK (Extracellular signal-regulated kinases); p-ERK (Phosphorylated ERK); L-NAME (NG-nitro-l-arginine methyl ester); nNOS (Neuronal nitric oxide synthase); MAPK (Mitogen-activated protein kinase)

The ERK signaling is modulated by NO in a way that it controls the activation and phosphorylation of ERK as studied in neuron-derived cell lines (Meini et al., 2006[45]) and in retinal and glial cells (Socodato et al., 2009[62]). Another research evaluated the role of nNOS on the activation of ERK due to the high dose of morphine treatment (Komatsu et al., 2007[33]), while the ERK inhibitor attenuated the expression of nNOS induced by the ERK activation in morphine withdrawal (Cao et al., 2006[8]). By considering that Ras-ERK cascade is needed for nNOS induction (Schonhoff et al., 2001[59]), the possible interaction of CaMK11 and nNOS through the activation of ERK was studied which increased NO by chronic morphine tolerance in harvested dorsal root ganglion (Wang et al., 2011[69]). The results of the present study reinforce the hypothesis that NO is significantly decreased by CsA, and that this decrease probably down-regulates the chronic morphine-induced phosphorylation of ERK1/2. Our hypothesis was further confirmed when we observed that nNOS protein expression was up-regulated by the phosphorylation of ERK1/2 in the morphine-dependent cells, and was down-regulated by CsA, which in turn suppressed the formation of the morphine-induced NO through a feedback mechanism (Figure 7(Fig. 7)).

In conclusion, the findings of the present study clearly demonstrated that cyclosporin A attenuated morphine-induced tolerance in the T98G cells, through the significant reduction of the levels of cAMP and by acting through NO/ERK signaling pathway. These findings suggest a novel approach in the treatment of morphine tolerance, and provide better therapeutic options for the clinical use. The results of the present study are significant from many aspects. However, further analyses are required to evaluate and confirm the underlying mechanisms with regard to the role of Cyclosporin A in morphine tolerance.

## Acknowledgement

As part of a Ph.D. dissertation, this project was financially supported by Experimental Medicine Research Center, Tehran University of Medical Sciences, Tehran (Grant number: 96-01-30-34351).

## Conflicts of interest

The authors declare that in this study there are no conflicts of interest.
